# National Neuroinformatics Framework for Canadian Consortium on Neurodegeneration in Aging (CCNA)

**DOI:** 10.3389/fninf.2018.00085

**Published:** 2018-12-21

**Authors:** Zia Mohaddes, Samir Das, Rida Abou-Haidar, Mouna Safi-Harab, David Blader, Jessica Callegaro, Charlie Henri-Bellemare, Jingla-Fri Tunteng, Leigh Evans, Tara Campbell, Derek Lo, Pierre-Emmanuel Morin, Victor Whitehead, Howard Chertkow, Alan C. Evans

**Affiliations:** ^1^McGill Centre for Integrative Neuroscience, Montreal, QC, Canada; ^2^Montreal Neurological Institute, Montreal, QC, Canada; ^3^Centre de recherche de l'Institut Universitaire de Gériatrie de Montréal, Montreal, QC, Canada; ^4^Lady Davis Institute, Montreal, QC, Canada; ^5^Department of Neurology and Neurosurgery, McGill University, Montreal, QC, Canada

**Keywords:** database, neuroimaging, infrastructure, dementia, Alzheimer's

## Abstract

The Canadian Institutes for Health Research (CIHR) launched the “International Collaborative Research Strategy for Alzheimer's Disease” as a signature initiative, focusing on Alzheimer's Disease (AD) and related neurodegenerative disorders (NDDs). The Canadian Consortium for Neurodegeneration and Aging (CCNA) was subsequently established to coordinate and strengthen Canadian research on AD and NDDs. To facilitate this research, CCNA uses LORIS, a modular data management system that integrates acquisition, storage, curation, and dissemination across multiple modalities. Through an unprecedented national collaboration studying various groups of dementia-related diagnoses, CCNA aims to investigate and develop proactive treatment strategies to improve disease prognosis and quality of life of those affected. However, this constitutes a unique technical undertaking, as heterogeneous data collected from sites across Canada must be uniformly organized, stored, and processed in a consistent manner. Currently clinical, neuropsychological, imaging, genomic, and biospecimen data for 509 CCNA subjects have been uploaded to LORIS. In addition, data validation is handled through a number of quality control (QC) measures such as double data entry (DDE), conflict flagging and resolution, imaging protocol checks^1^, and visual imaging quality validation. Site coordinators are also notified of incidental findings found in MRI reads or biosample analyses. Data is then disseminated to CCNA researchers via a web-based Data-Querying Tool (DQT). This paper will detail the wide array of capabilities handled by LORIS for CCNA, aiming to provide the necessary neuroinformatic infrastructure for this nation-wide investigation of healthy and diseased aging.

## Introduction

With 500,000 Canadians diagnosed with Alzheimer's Disease (AD), neurodegenerative diseases (NDDs) are becoming an increasing priority for Canadian society due to their significant and increasing socio-economic costs, which is estimated nationally at 15 billion Canadian dollars annually, and expected to rise to 150 billion dollars by 2038 (Fostering Alzheimer Society of Canada, 2010; Statistics Canada, 2016). As a result, conducting a nationwide study to investigate NDDs is paramount for achieving a better understanding of their etiologies, finding ways to mitigate their impact, and ultimately preventing their development. In response, an initiative spearheaded by the Canadian Institutes of Health Research (CIHR)^2^ and supported by various provincial and non-governmental partners (Appendix 1), assembled 340 researchers from across Canada to form the Canadian Consortium on Neurodegeneration and Aging (CCNA)^3^ The ultimate mandate of CCNA is to coordinate and strengthen Canadian research groups to better delineate and manage the causes, early detection, and treatment of dementia.

The primary vehicle for pursuing this mandate is the Comprehensive Assessment of Neurodegeneration and Dementia (COMPASS-ND), the signature clinical study of CCNA, which is currently collecting clinical, sensory, neuropsychological, neuroimaging, biological, and genetic data from a cohort of 1,650 individuals aged 50–90 with multiple types and severities of cognitive impairment, as well as 660 cognitively intact elderly individuals, recruited across 30 Canadian sites (Appendix 2). This study poses a unique and challenging technical undertaking, as it requires curation and standardization of diversified data from numerous sites across the country. To this end, CCNA has deployed LORIS^4^, a web-based data management system for multi-site studies, to facilitate collection, processing, analysis, and dissemination of multi-modal data, while ensuring accuracy and completeness with numerous quality control (QC) metrics in place (Das et al., 2012, 2016). LORIS has been tailored to CCNA's needs through the customization of key features, such as (1) Bilingual forms, (2) Training Portal for the familiarization of clinical and neuropsychological measures (Campbell, 2017), (3) Study Tracker for detailed study progression, 4) Biobanking module with support for any type of biospecimen, (5) Genomic Browser hosting CPG, SNP, and CNV genomics data (Rogers, 2015), (6) Imaging Uploader for multi-modal imaging data (Mohaddes, 2015), (7) Radiological Review module for incidental finding alerts and tracking, (8) Web-based Data-Querying Tool (DQT) that enables customizable query-building and data extraction (MacFarlane, 2014), (9) Configuration module to allow end-users to customize the interface, and (10) Publications module to manage consortium-led publications. LORIS' user-friendly interface, visualization tools, and targeted workflows also conveniently connect interdisciplinary teams of researchers on one platform.

In designing the specific features of LORIS for use within CCNA, many lessons from other international initiatives have been considered. Firstly, emphasis has been placed on building features within LORIS that are FAIR (Findable, Accessible, Interoperable and Reusable) (Chertkow, under review). The driving impetus revolves around concerns of usability and accuracy of data, especially given that the field of neuroscience (among other scientific fields) has been faced with a “reproducibility crisis” (Bennet and Miller, 2010; Glatard et al., 2015; Eklund et al., 2016; Fostering reproducible fMRI Research, 2017; Gilmore et al., 2017). As data proliferates, the methods of managing this data need to be carefully considered to avoid time and resource-consuming errors related to the increased order of complexity in its handling.

Through the use of a comprehensive data management system, CCNA hopes to contribute pivotal research findings to expand our understanding of NDDs, working toward prevention, and improving the quality of life for those with dementia. This paper examines how LORIS is tailored to the specific workflows within CCNA and highlights key features that have been implemented in order to facilitate data sharing between CCNA and similar studies at a provincial level, including Ontario Neurodegenerative Disease Research Initiative (ONDRI)^5^ and Consortium for the Early Identification of Alzheimer's Disease (CIMA-Q)^6^

## Methods

The CCNA infrastructure is composed of numerous elements that need to be organized, interoperable, and scalable, while conforming to the CCNA-specific cohorts, such as COMPASS-ND, the 5-years observational study aimed at recruiting 1,650 subjects (Chertkow, under review). The LORIS platform has been chosen to service this national initiative and has been set up to: (1) enable clinical sites to collect behavioral data with multi-modal QC checks, and direct feedback to data entry personnel, (2) facilitate biosample collection, (3) streamline imaging acquisitions and related QC metrics, (4) allow data sharing among researchers by performing self-administered queries, while tracking participant status and data entry progress throughout the study, (5) improve interoperability between different projects using APIs, and (6) provide comprehensive user support for the CCNA research community. As highlighted below, we will examine each of these aspects to delineate how the various multi-modal workflows have been configured in LORIS.

### Behavioral Data Acquisition

Behavioral data acquisition includes the collection of numerous measures (clinical, demographic, psychometric, and neuropsychological) from subjects across study sites. Data are collected through the use of paper forms which are later digitized by entering the information into replicate online forms. Several QC steps have been implemented in order to prevent the storage and distribution of erroneous information at the time of dissemination. Rules are also coded within the LORIS forms to ensure the completion of all required fields, avoiding accidental omission of critical data. Similarly, fields that are not contingent on the condition of a particular response are exempt to avoid redundant data. Forms are also translated in both French and English, with a unified backend storage solution that stores a singular value across languages for analysis consistency.

**Figure 1:**
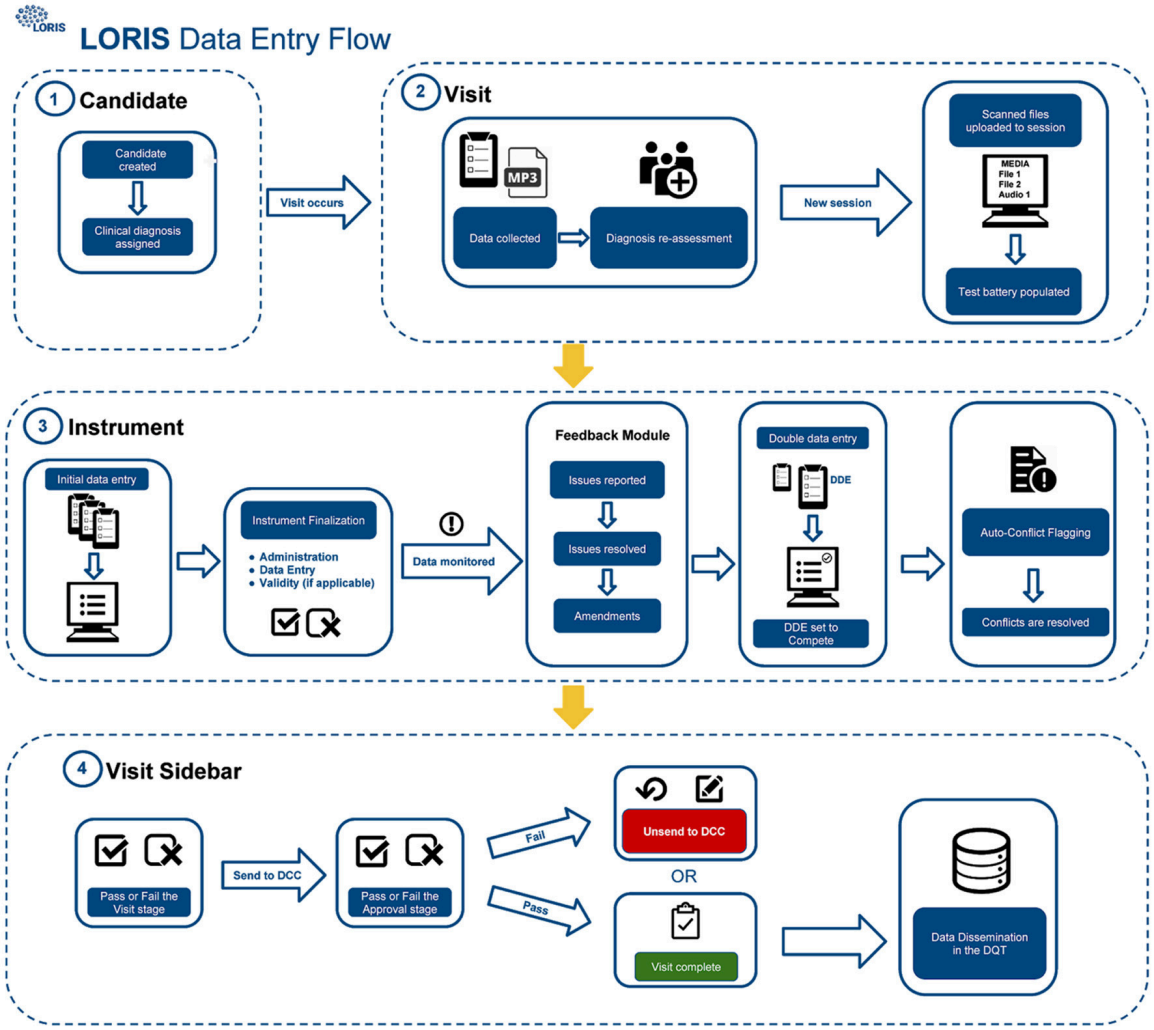
CCNA data-upload flow in LORIS. Step 1: Creation and registration of subjects, Step 2: Creation of timepoint and media upload, Step 3: Behavioral data entry and QC, and Step 4: Visit review and data dissemination.

The procedure for uploading participant data into the database (Figure 1) includes four different QC checks: (1) double data entry (DDE), (2) conflict resolution, (3) monitoring, and (4) final submission validation. As described in Figure 1, scanned documents and audio files are uploaded to LORIS via the Media Uploader where COMPASS-ND^7^ naming conventions are imposed. Subsequently, data monitoring checks take place between initial and DDE. Once data have been validated and pertinent details have been reported by a monitor via the Behavioral Feedback tool, DDE is then completed by a second individual. In case of a discrepancy between initial and DDE results, a conflict will automatically be flagged in the Conflict Resolver module. Conflicts are then resolved when data entry personnel review and manually select the correct answer from the conflicting options, by cross-referencing the scanned original paper forms (uploaded to the Media Uploader).

Finally, once all conflicting data have been amended and behavioral feedback have been addressed, forms can be set to “Complete” and the timepoint can be submitted for final validation. This final step requires a specialist to confirm that all necessary actions were taken and that the data are correct and ready for release; only at this point will the data be frozen to avoid any further modifications, and made available for dissemination.

### Biosample Collection

**Figure 2:**
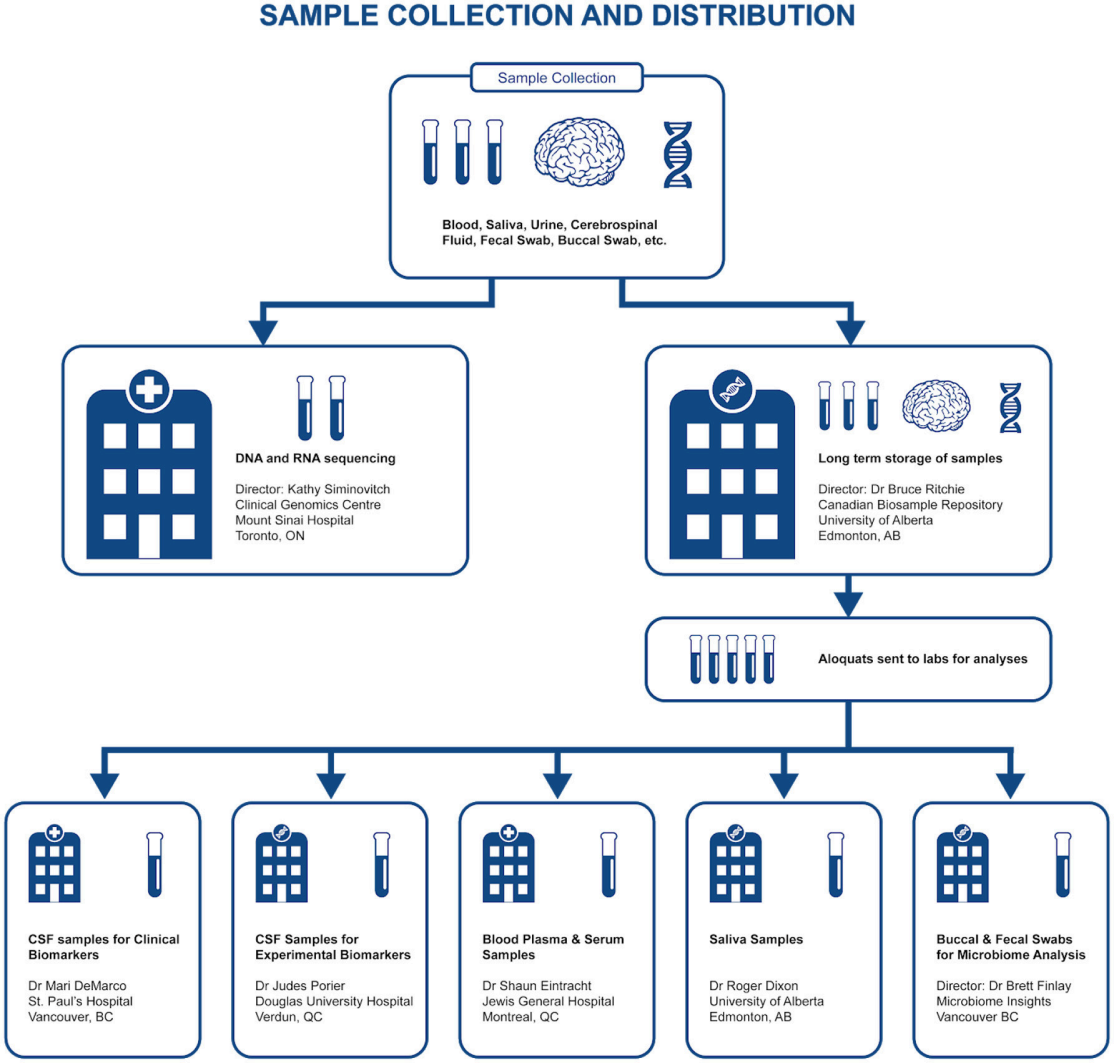
Sample collection and distribution.

Representing a core aspect of the data collected for the COMPASS-ND Study, biospecimen collection brings its share of challenges to the project. Biosamples collected from a subject can not be digitized and archived in the same manner as imaging and behavioral data, because the sample location, status, and availability must be easily accessible while continuously staying up to date, both in the system and in their physical storage. A Biobanking tool within LORIS was designed specifically for these purposes. Each collected specimen has a predefined storage destination (see Table 1) and processing plan (see Figure 2). CCNA draws on expertise from researchers across Canada including, but not limited to, the Toronto Genomics Center and the Canadian Biosample Repository (CBSR). As the CBSR facilities provide a biosample storage solution for CCNA's needs, LORIS adapted its sample tracking tool to streamline interactions with the CBSR database. This collaborative effort has led to the development of a strict standard for export and import of biospecimen data between CCNA sites and the CBSR database, reinforced on both ends by LORIS software and CBSR-Biobank software, respectively.

**Table 1:** Sample types, amounts, and destination.

**Sample type**	**Amount (tubes)**	**Amount**	**Destination**
Whole blood	6 ml (two 3 ml tubes)	N/A	Sent to local labs for CBC and HbA1C analysis
Serum	30 ml (three 10 ml red top tubes)	15 ml	30 aliquots → CBSR
Buffy Coat	12 ml (two 6 ml lavender top tubes)	1 ml	2 aliquots → CBSR
Plasma		6 ml	12 aliquots → CBSR
DNA	6 ml (one 6 ml lavender top tube)	N/A	Genomics Center, Toronto
RNA	2.5 ml (one 2.5 ml paxgene tube)	N/A	Genomics Center, Toronto
Urine	Up to 60 ml	6 ml	12 aliquots → CBSR
Saliva	4 ml (1 DNA genotek tube)	4 ml	8 aliquots → CBSR
			3 ml → locally for glucose, cells
CSF	15 ml (two 10 ml polypropylene tubes for 12 ml plus two standard hospital tubes 1–2 ml each)	15 ml	24 aliquots → CBSR

### Imaging

**Figure 3:**
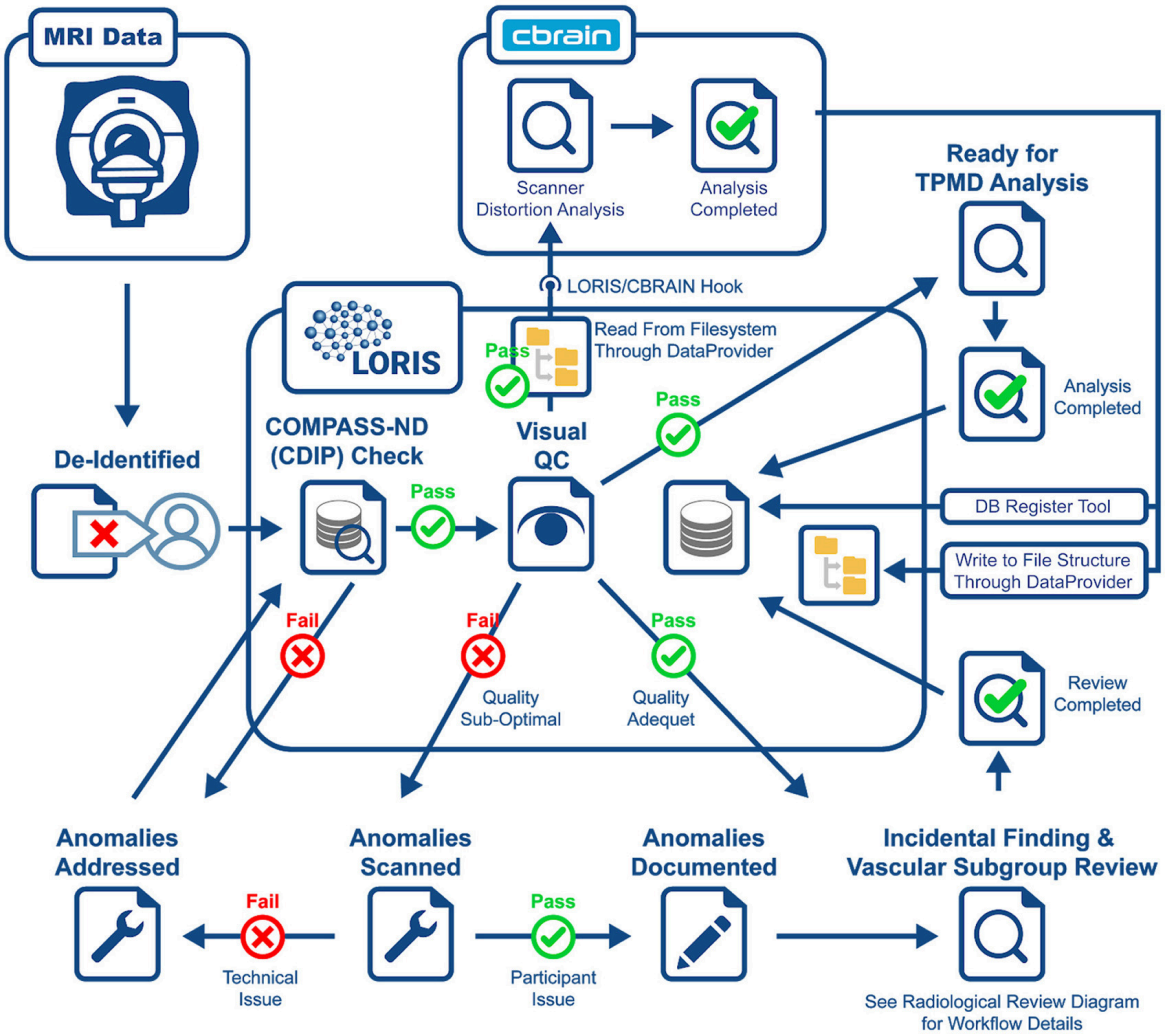
Imaging workflow.

Another crucial aspect to CCNA's COMPASS-ND project is the neuroimaging collection. The imaging workflow has been summarized in Figure 3 and consists of (1) scan collection, (2) visual QC, (3) MRI reads, and (4) storing volumetric data analysis derived from external tools. An important step in this workflow is the automated notifications to the sites when necessary.

#### Scan Collection

A major aspect of a multi-site study like COMPASS-ND is the importance of timely scan upload by the acquisition sites. Enabling faster QC allows for re-scan possibilities and quicker response to incidental findings. Furthermore, the COMPASS-ND central administration has set up a site reimbursement policy whereby scan costs are reimbursed only after images have been uploaded to LORIS and have adequate quality based on visual assessments. This has resulted in reducing (if not eliminating) missed scan uploads.

Another challenge in the success of this multi-site study is adherence to a common MRI protocol that is flexible enough to accommodate different scanner hardware capabilities across manufacturers (Siemens, GE, and Philips), yet strict enough to maintain a certain level of parameter uniformity across the different MRI modalities collected. The Canadian Dementia Imaging Protocol (CDIP; Duchesne, 2015; Duchesne et al., 2018) used in CIMA-Q, a preceding dementia study in Québec (Belleville, 2014), was extended to cover an increased number of scanners (7) in CIMA-Q vs. 20 in CCNA) and recruitment sites (7) in CIMA-Q vs. 35 in CCNA), thereby striking the balance between flexibility and uniformity.

#### Visual QC

All scans inserted into LORIS are reviewed visually by a trained team looking for artifacts (motion, scanner, intensity, and others). Newly inserted scans can be identified quickly which facilitates the visual QC feedback to be entered directly in the system. The goal of this step is to identify images within a session that pass QC standards set by the study, allowing further analysis to proceed. In addition, in failed QC cases, further information is communicated to the site to decide on the proper course of action (subject re-scan if required).

#### Incidental Findings

**Figure 4:**
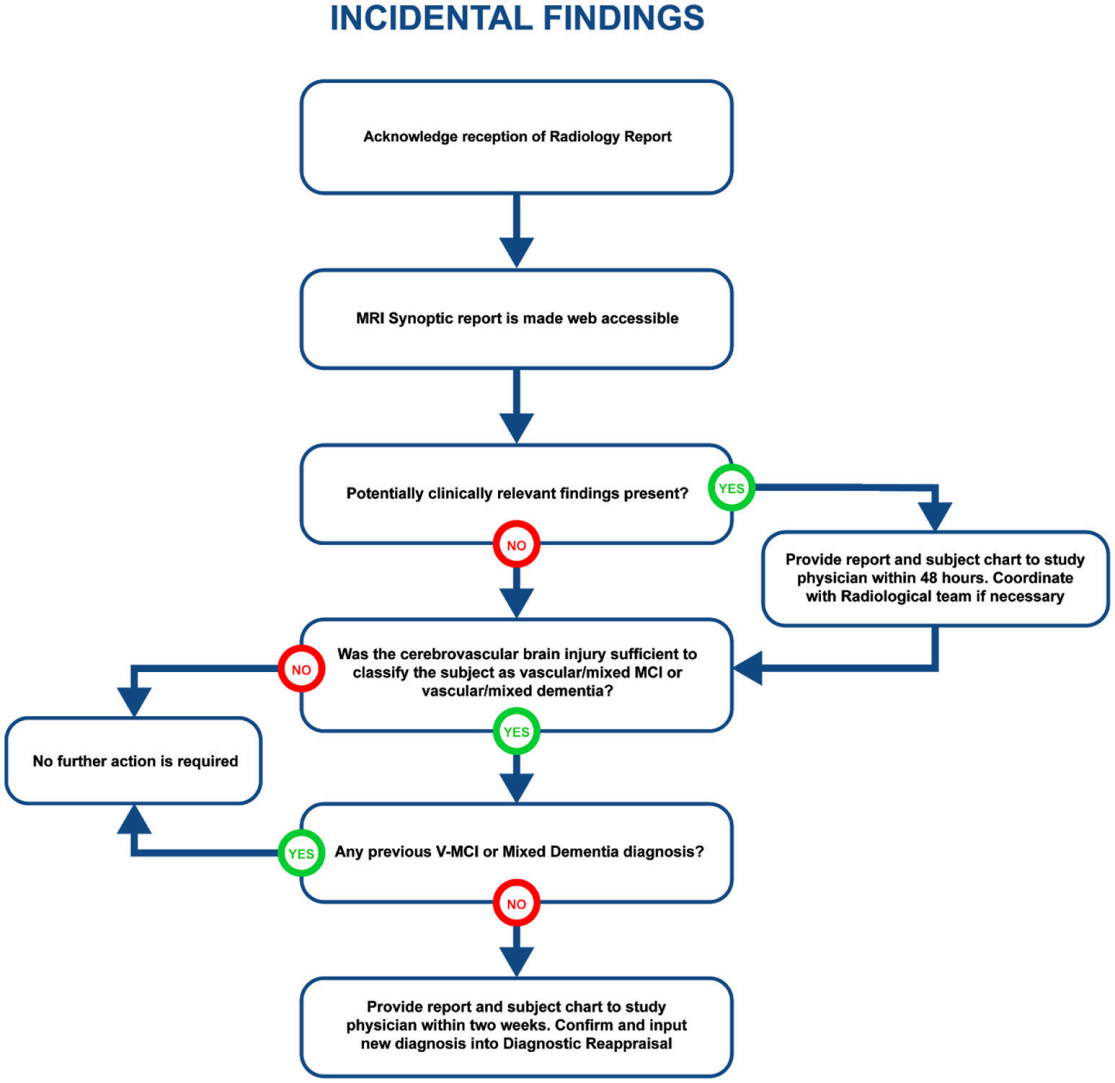
Detailed diagram of procedure for incidental findings.

Visually QC'ed sessions are important because they trigger another important step in the imaging workflow, namely, the MRI interpretations by another specialized team. The goal is to identify any incidental findings and report back to the site coordinator for further action in a timely manner. Forms with restricted access granted to the COMPASS-ND radiology team are devised for the study, and a notification system to alert the site when this “research” read is completed. This notification system points the site coordinator to a text printable version of the report, and requires an acknowledgment of receipt of such notification by the site. In case of incidental findings, the site takes the full responsibility to follow up with the subject's physician, while LORIS facilitates physician access to the images, if desired. A detailed workflow diagram and timeline is shown below (see Figure 4 for incidental findings procedure).

#### Derived Data

Although derived data of non-imaging modalities are being incorporated, the focus here is on illustrating how two types of derived imaging data, both constituting an integral part of the COMPASS-ND imaging workflow and mandate, are being integrated back to LORIS. The first is an automated hippocampal segmentation analysis based on True Positive Medical Devices (TPMD)^8^, where the goal is to perform volumetric analysis, along with z-scores on different brain regions in comparison with a control cohort from the Alzheimer's Disease Neuroimaging Initiative (ADNI^9^). LORIS is extended to facilitate this task using a set of tools that imports this data, processed externally, back onto LORIS. The larger goal of importing this data back into LORIS as behavioral forms is made possible through built-in DQT capabilities that render this information as easily queryable and accessible by researchers.

The second type of derived data is the lego phantom image processing results (Fonov et al., 2010) for scanner distortion identification and parameter extraction. These data are re-inserted into LORIS for further correction by researchers, if needed. A LORIS-CBRAIN hook was developed to read phantom images (T1W, T2W, PD, and resting state fMRI) from LORIS' filesystem, processed on CBRAIN (Sherif et al., 2014), with processed images and files put back onto the COMPASS-ND filesystem. This used a containerized version of the Fonov processing pipeline. Automatic launching of these tasks directly from LORIS (i.e., without requiring logging into and launching the task from within CBRAIN) is currently being developed, making the process completely automated to the LORIS CCNA user.

### Data-Dissemination

Data dissemination is a crucial aspect in processing and analysis. For this reason, we have incorporated several tools into LORIS to facilitate querying, download and sharing, including our DQT and Study Tracker.

#### The Data Query Tool

Historically, researchers required a programmer or database administrator to query the database, and disseminate particular data outputs. This had potentially translated into days or weeks of delay for their investigations. Today, LORIS assigns great priority to data dissemination and enables researchers to directly query the database and easily retrieve data. The DQT allows researchers to design, execute, and save queries in a simple and intuitive manner, without having to write complex database queries. The interface allows for selection of variables, and quick download in most commonly used formats (e.g., Excel, comma separated values, and tab separated values). In addition, users can save any query (both the variables and the population) and use it in the future with new or updated data, without worrying about ambiguities and inconsistencies. Datasets can also be tagged with a version number or a timestamp such that longitudinal comparisons can be made, minimizing any ambiguity about what has been downloaded (Das et al., 2012; MacFarlane, 2014).

#### Study Tracker

**Figure 5:**
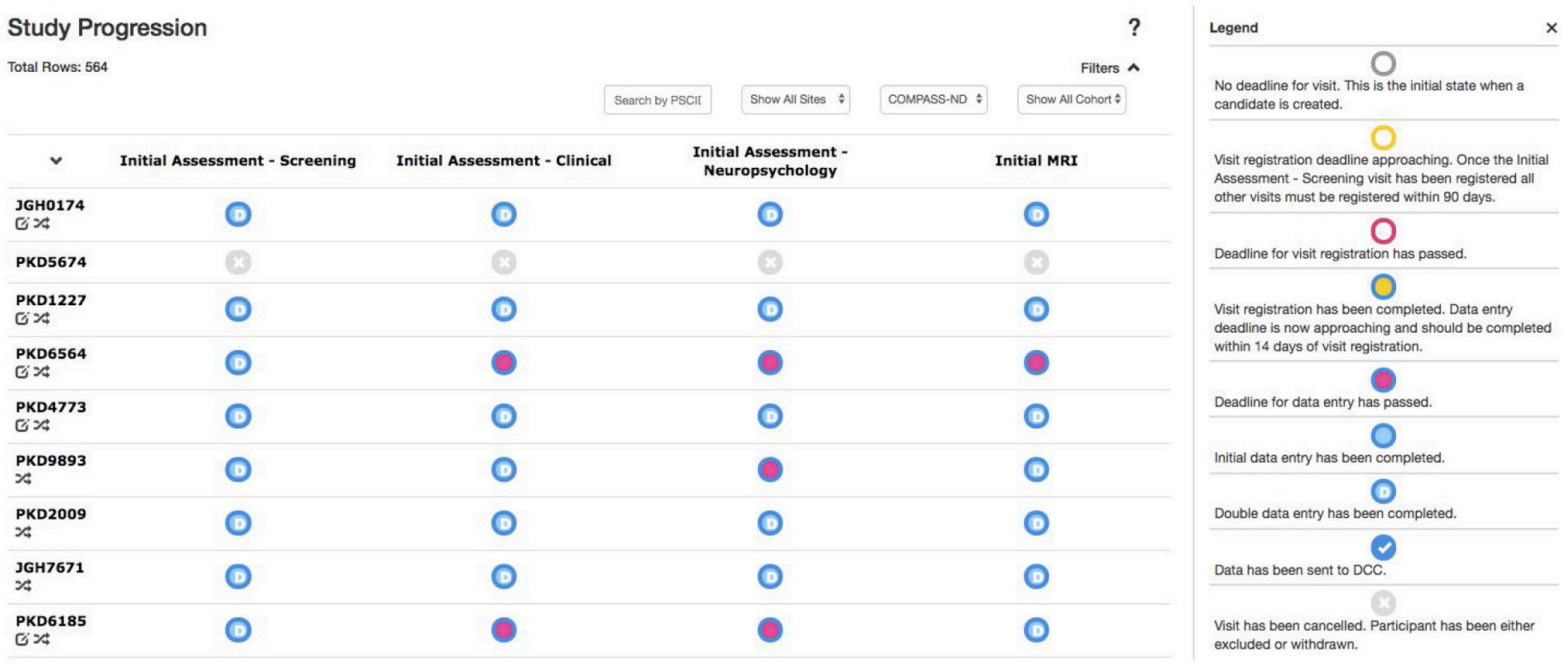
Study Tracker. Each row corresponds to a participant, with each circle in that row corresponding to a visit for that participant. The border color of the circle represents the status of visit registration and the fill indicates data entry completion with respect to the due date. Here the open sidebar has the visit specific view in focus, with links to individual forms as well as links to the Conflict Resolver.

Due to the magnitude and complexity of the COMPASS-ND project, there is substantial difficulty in monitoring the progress of data entry and visit registration among the dozens of study sites submitting hundreds of different forms. Queries to the database to acquire this information would be considerably complex, with outputs that are convoluted and time consuming for users to parse. With this in mind, the Study Tracker module was developed, as described in Figure 5, to provide study administrators an efficient graphical interface to view the progression of data entry and visit registration. Using a simple color coded system, representing data entry completion relative to each timepoint's individual deadline, users are able to quickly identify data entry bottlenecks and unresolved issues for each study participant at every visit. Focusing on a specific timepoint brings up links that allow users to navigate to: (1) individual measures administered at that timepoint, (2) Conflict Resolver module to settle conflicting data entry values, and (3) open Behavioral Feedback discussion threads.

### Interoperability

**Figure 6:**
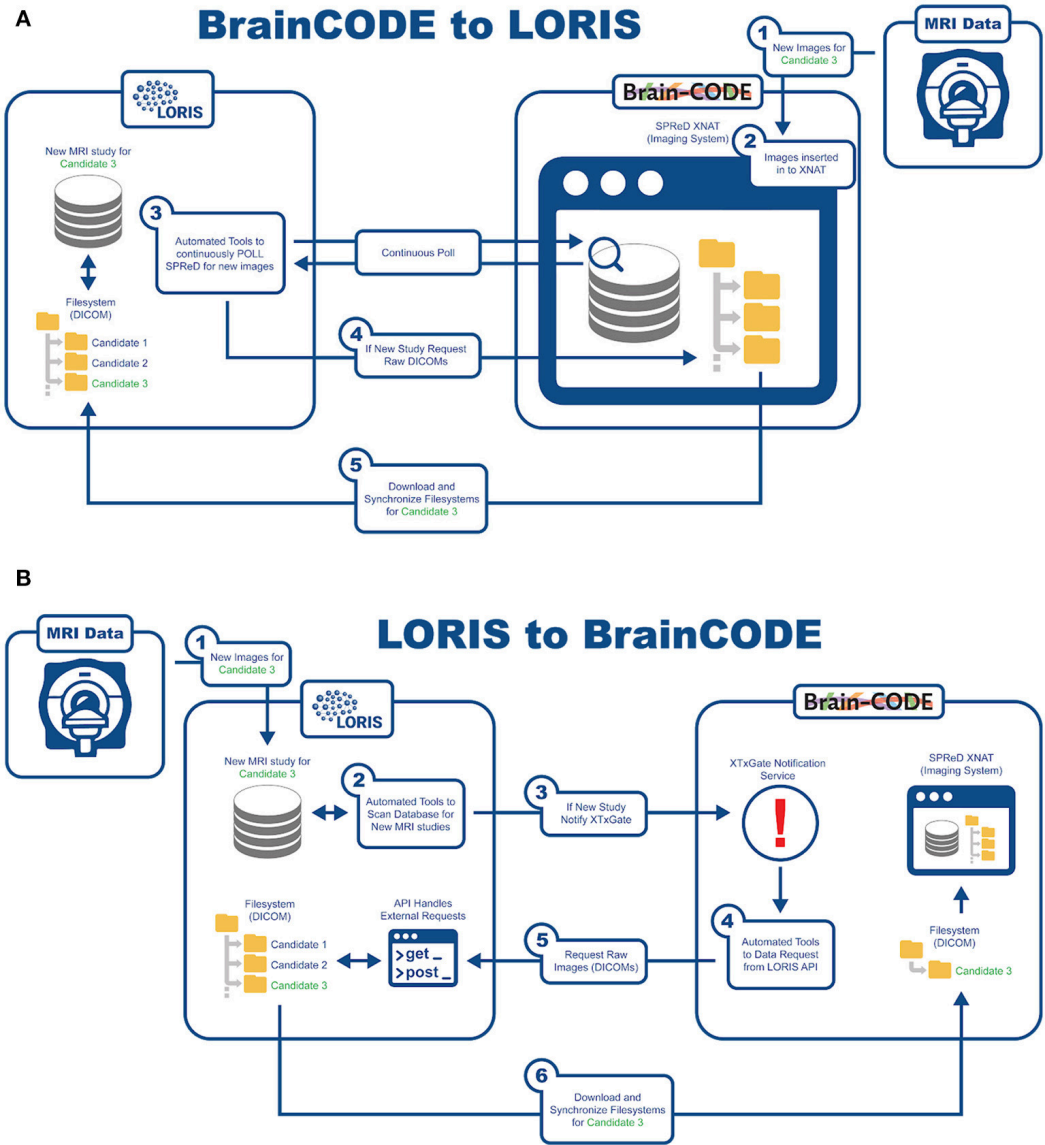
Imaging transfer **(A)** BrainCODE to LORIS **(B)** LORIS to BrainCODE.

An important aspect of this study is being able to aggregate data from already existing nodes. In order to reach COMPASS-ND's enrollment target, interoperability has been developed with BrainCODE^10^, a platform that houses the ONDRI study as part of CCNA's mandate. There are currently about 150 subjects in ONDRI that can be shared with CCNA. As such, LORIS and BrainCODE needed to customize their API capabilities to enable a 2-way transfer of imaging data that adheres to the internal structures and desired business rules of both systems. Transfer of images from BrainCODE to LORIS is based on LORIS polling BrainCode's XNAT imaging storage system through a middleware interface (Figure 6). Conversely, transferring images to BrainCODE relies on a LORIS initiation of an automated message to BrainCODE's XTXGate^11^ notification system. The XTXGate system receives a JSON-encoded message from LORIS with all metadata necessary to identify, and subsequently download, the raw DICOM images of the newly added MRI study using LORIS' API.

### User Support

Serving as a centralized user support portal for the consortium, the Member's Portal is a forum relying on the Discourse software^12^ to connect users, coordinators, and administrators to each other. The Discourse application is integrated inside LORIS to avoid confusion and the steeper learning curves associated with multiple-platform training. The portal posts are constantly and consistently monitored by moderators and support staff to ensure the content is appropriate and triaged in the appropriate categories.

## Results

One of the major goals of CCNA is to develop a national cohort to provide researchers with data to test and refine their hypotheses about the progression of dementia. LORIS itself is the tool through which the research community can access data in a trustworthy and robust manner. This section will highlight the data that were acquired, as well as several features that have been tailored and developed within LORIS.

### Behavioral Data in Numbers

Presently, CCNA has 509 registered subjects for COMPASS-ND across 10 cohorts of various NDDs, 459 (90.17%) of which are currently active in the study, omitting subjects who have since become inactive (excluded, ineligible, etc.). Table 2 summarizes the number of registered subjects by cohort and includes an in-depth breakdown of currently active subjects by sex. COMPASS-ND will enroll 1,650 participants with various types and severities of cognitive impairment, as well as 660 cognitively intact participants, which will be “deeply phenotyped” through data collection from numerous modalities (Chertkow, under review).

**Table 2:** Subject demographics per cohort.

**Cohort**	**Registered subjects total**	**Active subjects total**	**Male**	**Female**
SCI	82	76	23 (30.3%)	53 (69.7%)
MCI	186	176	86 (48.9.%)	90 (51.1%)
V-MCI	51	47	31 (66.0%)	16 (34.0%)
AD	71	55	34 (61.8%)	21 (38.2%)
Mixed	22	18	14 (77.8%)	4 (22.2%)
FTD	23	16	11 (68.7%)	5 (31.3%)
PD	21	29	18 (62.1%)	11 (37.9%)
PD-MCI	23	20	19 (86.4%)	3 (13.6%)
PDD	8	4	4 (100.0%)	0 (0.0%)
LBD	22	18	14 (77.8%)	4 (22.2%)
Total	509	459	252 (54.9%)	207 (45.1%)

*SCI, Subjective Cognitive Impairment; MCI, Mild Cognitive Impairment; V-MCI, Subcortical Ischemic Vascular MCI; AD, Alzheimer's disease; Mixed, Dementia of Mixed Etiology; FTD, Frontotemporal dementia; PD, Parkinson's dementia; PD-MCI, Parkinson's dementia (PD) with MCI; PDD, Parkinson's Disease Dementia; LBD, Lewy Body disease*.

Currently clinical, neuropsychological and biospecimen data for 509 COMPASS-ND subjects have been uploaded as described in steps 1–4 in Figure 1. Upon successful upload, COMPASS-ND coordinators use a suite of behavioral QC modules, such as the Conflict Resolver and Feedback Module, to ensure completeness and accuracy of the data. There have been just over 30,000 conflicts flagged since the launch of COMPASS-ND, spread over ~200 subjects with over 8,100 data fields each; this number represents a 1.84% data entry error rate and 60% conflict resolution rate. Once data have passed the approval stage, they can be flagged as “ready for dissemination” and are then accessible through the DQT.

### Biobank

**Figure 7:**
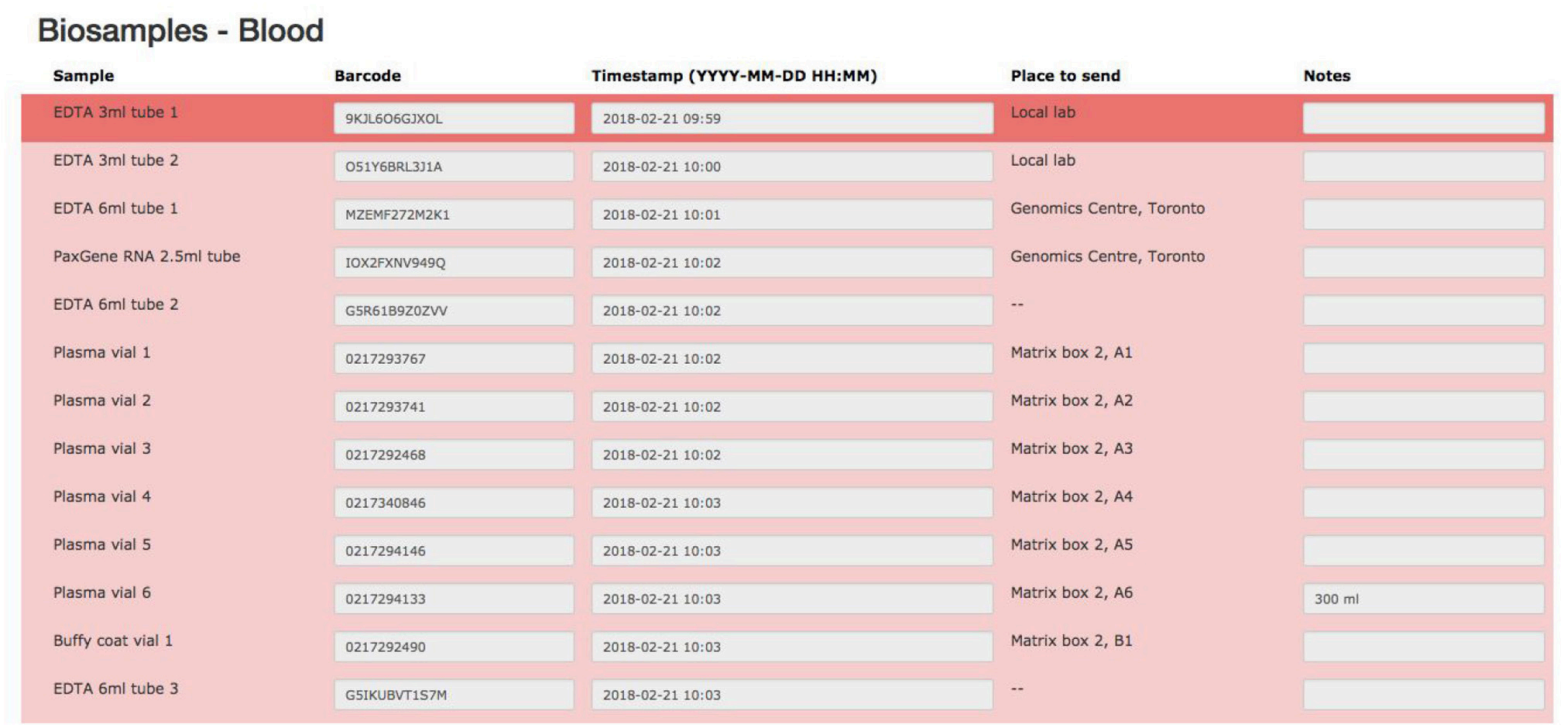
Biosamples collection forms.

The biospecimen collection and tracking needs for the study have been addressed by the development of a specialized tool in LORIS. This was implemented in order to streamline and increase the level of automation for acquisition, and tracking of biosamples on site during the subject's visit. The tool is composed of seven pages, each dealing with specific tissue types or bodily fluids organized in their order of collection: blood, urine, saliva, cerebrospinal fluid (CSF), buccal and fecal samples, and a final page for extras (if there are any unused vials). Each page is fully independent and each row on the page contains information on a single sample only (see Figure 7); a row contains information on the time of collection, barcode ID, destination and location of the biosample. Each field can also be saved independently. Furthermore, a set of rules and validation steps automatically enforced by the software prevent loss of data integrity. These rules ensure that all barcode IDs are unique in the database, that the barcode scanned is of the correct type for the specimen, that any modification is logged with a timestamp, and that no field is left incomplete. After all the biosamples are scanned into LORIS, a shipment flag is enabled indicating that the sample is ready to be sent to the off-site biospecimen repository; this flag can only be set if all validations pass. Before the samples are shipped out and the data are exported, a last quality check consisting of all of the validations above is run by an administrator on the data. This ensures that the exported data have not been modified between the entry date and export date. Through training of the staff, in conjunction with LORIS monitoring for omissions or errors in the data entry of biosamples, the risk of inconsistencies in the database is significantly reduced.

### Imaging in Numbers

A total of 467 imaging scans (364 subjects and 103 phantoms, 76 of which are for lego phantoms), conformant to the Canadian Dementia Imaging Protocol (CDIP), have been uploaded from 20 different sites via the Imaging Uploader (Mohaddes, 2015). Visualization and QC of image files are tailored to each modality and customized per type of review (on-site, centralized, manual, or automated). Of the 364 uploaded scans, 346 underwent visual QC on the different MRI subtype structural modalities (with the following visual QC failures: 6 T1W, 3 2D Flair, 17 T2^*^, and 5 dual echo PD/T2), and 331 were read for MRI incidental findings, of which 19 had potentially clinically relevant incidental findings. The remaining scans are queued to be QC'd as well, as our QC team manages to complete on average roughly a dozen QCs a week. In addition, TPMD processed data analysis for 266 subjects have been performed offline and results were imported back to LORIS, with more analyses from the remaining subjects to come. Each analysis contains specific anatomical measurements for gray and white matter volume and other quantitative biomarkers. Follow-up scans obtained from these subjects can then be processed using TPMD, allowing for not only the course of the degenerative diseases to be tracked, but quantified with precise measurements.

### Data Dissemination

**Figure 8:**
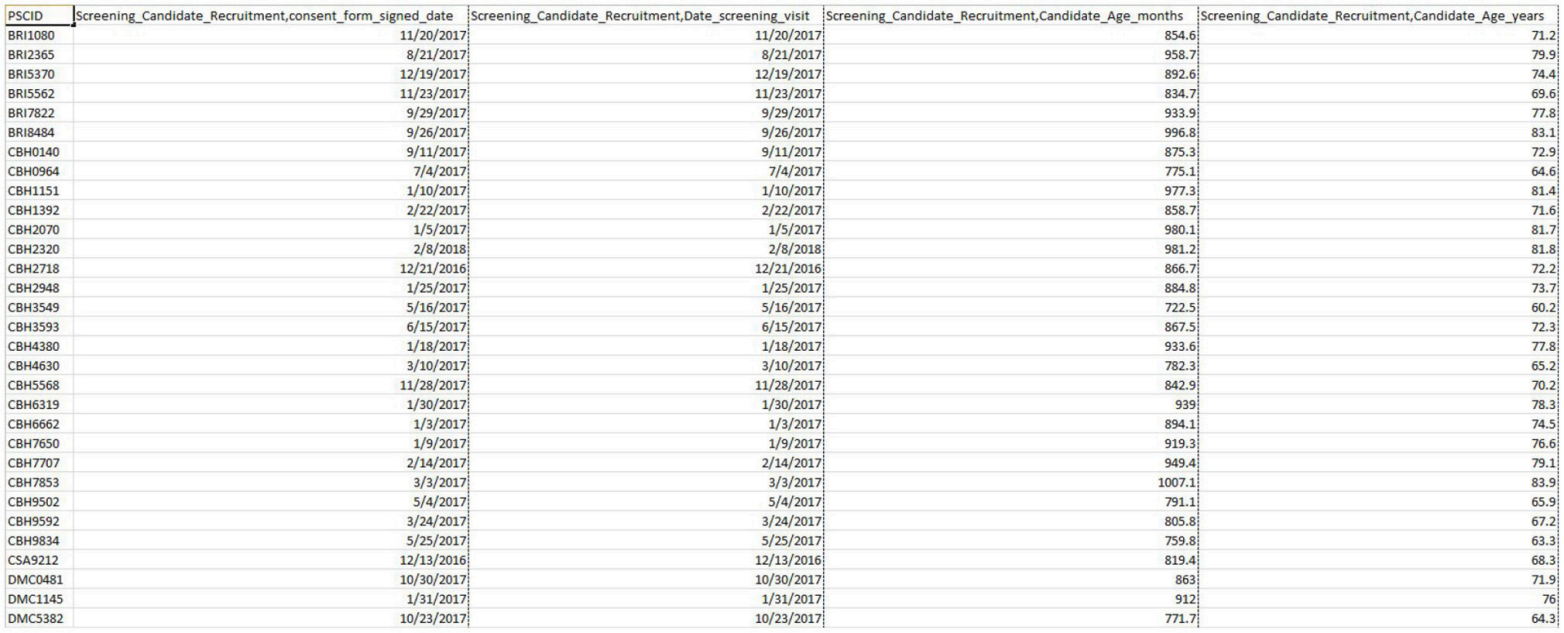
Sample result (extracted from DQT) for the upcoming CCNA release.

The web-based DQT enables download of validated scalar data (clinical, demographic, psychometric, biosample, and neuropsychological data) linked to imaging data through a simple querying interface (see Figure 8). Currently, curation has been completed on the first 4 initial assessment visits (screening, clinical, neuropsychological, and MRI) including data entry feedback and conflict resolution in preparation for an upcoming data release (fall of 2018) of the first 200 CCNA participants. This release for CCNA researchers will include collected and derived measures, all made accessible through a simple query in the DQT.

It is expected that this release will provide data to 17 different CCNA teams aiming at addressing 106 proposed research projects. Example questions include:

- What distinguishes V-MCI from MCI? Mixed from AD?
- How does frailty impact cognition?
- How does sensory impairment relate to cognitive impairment?
- Does a comprehensive blood profile distinguish MCI from AD?
- Do gait metrics distinguish AD from other types of dementia?

### Interoperability

The technical infrastructure for imaging interoperability between COMPASS-ND and ONDRI operates without user intervention. It consists of scan imports from ONDRI, and includes DICOM header de-identification, automatic file renaming, as well as insertion via the Imaging pipeline using customizable MRI protocol validation. In addition, the LORIS API was extended to allow sharing/downloading of raw DICOM images (along with its already existing MINC sharing capability). The addition of an on-demand notification system to inform ONDRI of any newly added DICOMs also allows for the flow of images from CCNA to ONDRI, facilitating bi-directional MRI scan exchange in a seamless manner.

### User Support in Numbers

User support in a project of this scope does require significant support mechanisms. The central purpose of the Members Portal is to provide user support, similar to a web forum where discussion occurs, and the highest number of posts are prioritized. Currently there are 457 support requests from users with 437 resolved issues, 12 with fixes underway, and 8 requiring more information from the reporter.

**Figure 9:**
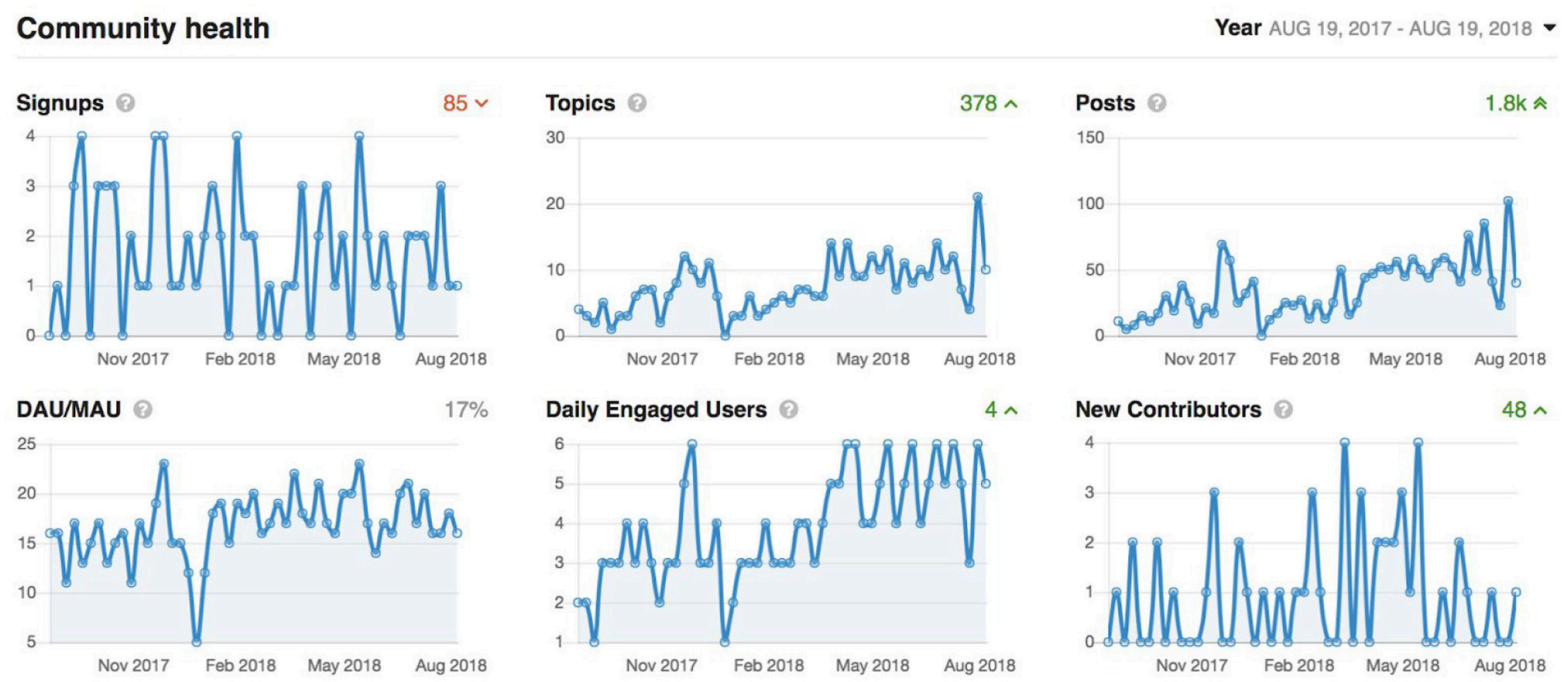
Graphs showing overall activity on the Member's Portal over the last year. Signups: accounts added which are imported automatically from the LORIS users list. Topics: number of new support requests. Posts: number of support replies from our user support team. Daily active users/monthly active users (DAU/MAU): number of members that logged in within the last day, divided by number of members that logged in within the last month. Daily Engaged Users: number of users that liked or posted something new per day. New Contributors: number of users that made their first contribution during the indicated period.

**Figure 10:**
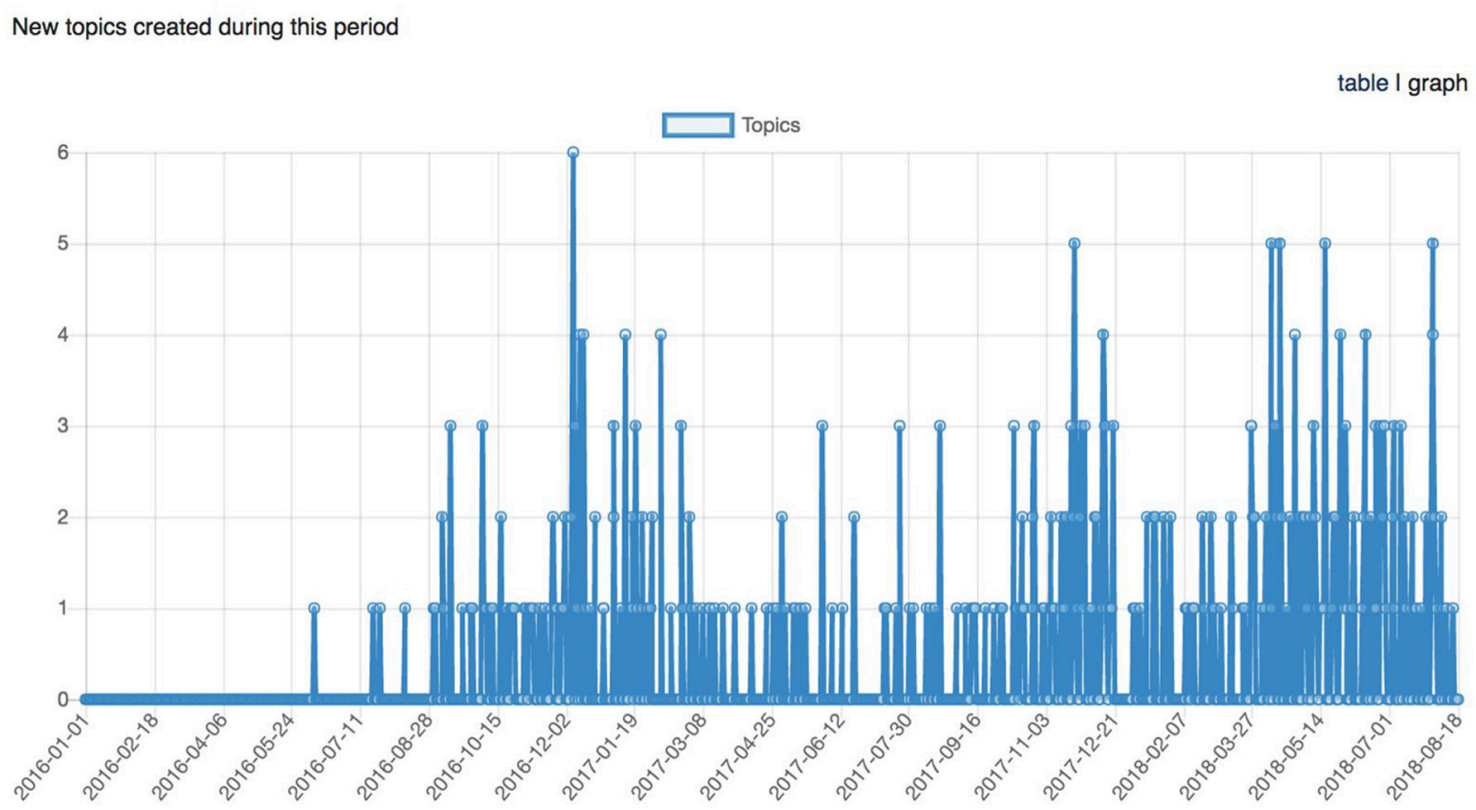
Graph representing the number of topics (support requests) created across time between January 1st, 2016 and August 18th, 2018.

**Figure 11:**
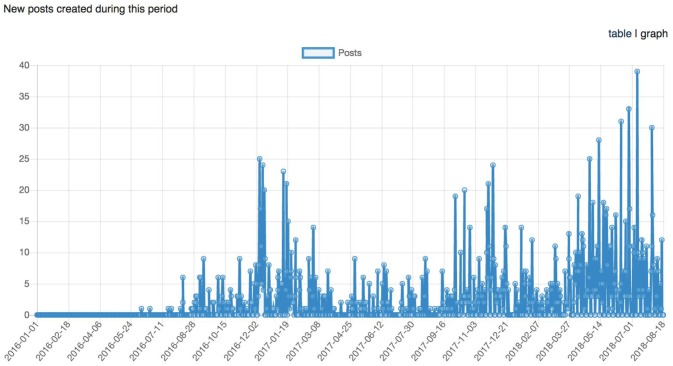
Graph representing the longitudinal number of posts (support request replies) between January 1st, 2016 and August 18th, 2018.

It has been nearly 2 years since the start of CCNA's COMPASS-ND data collection, and LORIS' technology and tools have been extended to fulfill COMPASS-ND's clinical and research needs. This is further demonstrated with a comprehensive, organized, and multi-modal dataset for 200 subjects that will be disseminated to CCNA researchers in fall 2018 (see Figures 9–11).

## Discussion

A national infrastructure for data management serves many needs that are paramount to the consortium's success (Toga, 2012). Having a tested technical platform for incoming data reduces the burden on researchers to manually manage data, and also provides tools and methods for accurate and efficient data entry (Poline et al., 2012; Nichols et al., 2016).

One of the most fundamental aspects is standardization, which remains an ongoing issue for any data sharing consortium. If data are properly structured, collaborative efforts become much more efficient and reduce the future burden of cross-site analysis (Gorgolewski et al., 2016; Munafo et al., 2017). To that effect, the CCNA infrastructure has incorporated many such standardization techniques in LORIS. Behavioral measures are coded in a structured manner, with all QC checkpoints rigorously enforced, and in turn, queryable alongside individual data items. Imaging acquisitions have been harmonized, as prescribed by the CDIP protocol. Biospecimen data have been organized to conform to a standard established between CCNA and CBSR, allowing for easier collaboration and safer data transfers. Collecting data according to a standardized common protocol and organizing it in a uniform manner makes it easier to process and share, while simultaneously reducing error rates resulting from manual manipulation of data (Gorgolewski and Poldrack, 2016). A great problem in the neuroscience community has been the ability to properly reproduce findings, an issue that stems in no small part from the lack of consistency of the data (Zuo and Xing, 2014; Zuo et al., 2014; Turner et al., 2018).

A major challenge within a national network, such as CCNA is ensuring interoperability between all technical segments and institutions. This is achieved through standardization initiatives, ensuring data harmonization, and proper API documentation and development useable between existing platforms and tools. To that effect, the CCNA-LORIS system has demonstrated interoperability, in that LORIS and BrainCODE regularly exchange imaging data between the ONDRI study and the national CCNA data platform. The technical software infrastructure for bi-directional exchange of behavioral data can mirror the imaging data, as embedded API functionalities make this sharing of behavioral data possible (for example in JSON format). However, any such behavioral data exchange includes additional challenges, in large part due to the harmonization required to translate data forms and dictionaries between ONDRI and CCNA or any two projects in general (Richesson and Nadkami, 2011). An extensive data mapping exercise is currently underway in LORIS transferring behavioral data from CIMA-Q to CCNA, with a similar endeavor shortly taking place between ONDRI and CCNA. While LORIS includes a data dictionary tool to facilitate ontological harmonization for any given study, limitations persist to map ontologies across studies due to greater standardization issues within the neuroscience community.

Provenance capture is also an important requirement in ensuring that data are usable and reproducible. LORIS natively handles a great deal of provenance information. For example, images including any associated metadata (e.g., scanner specifications, protocols, demographic information, and image processing details) are always extracted and stored. Behavioral data, scoring, updates, corrections, and any QC results are also captured. In addition, project metadata, as well as a complete subject audit trail are always available. LORIS also leverages existing platforms to provide the necessary information required for analysis (Maumet et al., 2016), and works with standardization groups to ensure maximum provenance retention (Glatard et al., 2015; Gorgolewski et al., 2016)

Increasing sample sizes and recruitment targets can improve collaborations and result in reduced redundancy in how research resources are invested (Evans and Brain Development Cooperative Group, 2006). Nationally coordinated approaches to building infrastructure can ensure more fruitful returns to a greater number of researchers across federally-funded initiatives. Furthermore, increased population can result in a more diversified sample that considers demographic variability, which is key to generalizability of results (Zuo et al., 2014). These points are especially true when dealing with machine learning algorithms, which require access to large data samples that need to be organized in a consistent manner (Toga, 2012). In the absence of larger cohorts, researchers have traditionally leveraged smaller studies with non-standardized protocols for meta-analysis, often lacking adequate harmonization or normalization. Consequently, this results in significant variability and decreased confidence in findings. Momentum has accumulated toward sustaining a higher standard of data quality and volume to accelerate research discovery optimized for transparent and reliable results.

Establishing a unified methodological approach to data acquisition and processing can dramatically reduce the burden associated with calibration and harmonization (Poldrack and Poline, 2015). This setup typically requires less training as most researchers become familiar with one system for data management. Another benefit of a national infrastructure is the ability to leverage expertise of highly specialized laboratories for specific processing and analysis. Proven benefits of data sharing within a multi-site national infrastructure can be drawn from ADNI's global impact (Weiner et al., 2015). A decade since its inception, over 1000 scientific publications have been produced using this dataset (Toga and Crawford, 2015). There is a strong argument to be made that the choice of this approach has resulted not only in increased citations, but also in collaborations (Toga et al., 2017).

Despite the benefits, challenges persist in large data infrastructure and analysis (Kang et al., 2016). There is often an initial investment required in terms of effort to unify the various members of a scientific discipline, as well as a technical platform to develop (or adopt) a comprehensive infrastructure. The technical challenges can manifest themselves in a number of ways. Data management platforms need to be customizable, easily extensible, and highly interoperable. While LORIS has been designed with these features from its onset, establishing data access barriers between projects and sites continues to be improved. LORIS defines several access control for specific modules and modalities, however more granular permissions are being added (such as project-dependent access restrictions or project/site-specific permission assignment). Exchanging data between existing sites can require establishing common data definitions and exchange protocols. Often, specialized laboratories rely on external analysis tools that generate proprietary or unstandardized output, or in the case of neuroimaging analysis tools, rely on pipelines that are either developed in-house, or placed on distributed computing resources. It then becomes imperative for the data management system to carefully: (1) interact harmoniously with external tools in terms of not only reading, but also writing the data back onto the system, and (2) integrate analyzed and derived data in a harmonized and queryable manner.

Scaling these operations across a national consortium can also be an ongoing challenge. Leveraging the full power of high performance computing environments can involve a significant learning curve both at a low-level of computing infrastructure as well as higher level issues of processing and analysis (Da Mota et al., 2014).

Issues can also result from policy requirements that might differ between sites or regions. For any study, there are specific ethical and regulatory procedures that are governed by local ethics committee, regional governing boards, and legislative privacy and ethics laws. Although LORIS is not directly governed by these procedures, there are indirect consequences which constantly present new challenges to the software. A simple example of such procedures is amendments brought to existing instruments which go through ethics approval in each region of the country simultaneously, as they are reviewed independently in each province and approved at different times. During this process, sites in different provinces administer different versions of a single instrument, while LORIS, currently displays a single version of any instrument. Studies can certainly benefit from ethical frameworks for data sharing (Dyke et al., 2018) as these issues must be internalized when sharing data nationally (or internationally).

It should be noted that there are other systems that can curate and manage data, some of which have gained significant adoption, such as RedCap (Harris et al., 2009), a platform to create clinical and psychometric forms for web-based data entry. While this platform has been developed to simplify several aspects of data collection, it was not designed for multi-modal curation (such as imaging or genomics data). In comparison, LORIS is extensible, modular and scalable to allow for heterogeneous data types. There are numerous tools that manage parts of the curation process, but do not handle array of functionalities required for the full lifecycle of a longitudinal, multi-modal study. Other open source platforms also exist such as XNAT (Vaccarino et al., 2018) and COINS (Scott et al., 2011), each of which can handle such requirements, but have different methodologies for curation. While each of these systems could be leveraged, LORIS has recently heavily invested in open science principles and resulting data sharing capabilities.

Adhering to the *FAIR* principles (Findable, Accessible, Interoperable, and Reproducible) (Wilkinson et al., 2016) is a central tenet in building a data sharing platform (Gorgolewski et al., 2013). As CCNA continues to develop, the underpinning factors for successful data collection and sharing across a national consortia will likewise undergo enhancements, in supporting a technical infrastructure that is flexible enough to import data from other platforms, harmonize and coordinate data from different sources in a queryable manner, and seamless user-facing processes which increase the transparency and data management. To that end, CCNA has considered these concepts in its adoption of LORIS, which integrates and continues to enhance these facets in data sharing.

### Future Developments

Future developments are planned in the following areas: (1) biospecimen data, (2) imaging modalities, (3) new behavioral cohorts, and (4) CCNA-specific technological advancements.

1. Biospecimens: Collection will require the following features in the CCNA data platform:
  - *Biobank:* Processed data to be imported for subject biomarkers (general health metrics, sex-related hormones, inflammation, lipid metabolism, microbiome, and oxidative stress)
  - *Genomic Browser:* Extensive genotyping^13^ with summary epi/genetic data (CPG, SNP, and CNV) will be stored (and visualizable) to evaluate markers of genetic susceptibility.
  - *Brainbank:* Brain tissue pathology data for diagnosis and proteomics to be imported from Canadian ADNI Brain Donation & Neuropathology Network (Franklin et al., 2015).
2. Imaging: Upgrades are planned to incorporate additional modalities:
  - *Electrophysiological module:* COMPASS-ND to be extended to study epilepsy. LORIS will display EEG^14^, by integrating an EEG-BIDS^15^ reader for standardized EEG data.
  - *Positron Emission Tomography (PET):* Extensions to support PET from Siemens High-Resolution Research Tomograph (HRRT) is currently being developed.
3. Behavioral cohorts: Clinical/psychometric data will be coded in LORIS:
  - *Functional Assessment of Vascular Reactivity (FAVR)*^16^ Longitudinally investigating cognitive impairment and vascular dysfunction in AD, and small vessel disease for subjects with cerebral amyloid angiopathy, AD, and MCI.
  - *Normative Comparison & Control Group:* Cognitively intact older individuals, providing normative neuropsychological data to the COMPASS-ND battery.
  - *Sleep Study:* Identify brain mechanisms linking sleep and circadian rhythm disruption to cognitive decline and incident AD, and other dementias in older adults^17^.
  - COMPASS-ND Intervention Studies:
    ○ *SYNERGIC* (Synchronizing Exercise Remedies in Gait & Cognition)
    ○ *ENGAGE* (Exploring Novel Group Activities for Geriatric Enrichment)
    ○ *LEAD* (Lifestyle, Exercise, And Dementia).
4. Novel Technological components: New features and technologies enhance reliability:
  - Interoperability: DataLad and Git-Annex is currently being developed to:
    ○ Submit metadata and images from sites to a central repository.
    ○ Build multi-layered user-access with prolific levels of control for sharing data.
    ○ Leverage metadata searching capabilities already integrated within DataLad.
    ○ Seamlessly link metadata with the MRI images tracked through Git-Annex^18^.
    ○ Download images in BIDS format^19^ (Gorgolewski et al., 2016).
  - API: Automate time consuming tasks to improve interoperability/reliability with:
    ○ Direct database queries/updates to allow external apps to use LORIS.
    ○ Enable mass uploads.
    ○ Create subject identifiers.
    ○ Extract-Transform-Load tool to import data (SQL and JSON) with user-defined rules.
  - Standardization: To ensure reproducibility, several features can be improved:
    ○ Image processing containerization (e.g., tissue classification & volumetrics).
    ○ Common data elements are being leveraged increasingly.
    ○ Forms building tools^20^ to abet this process.
  - User Interface: Several updates will be made to optimize usability:
    ○ Dashboard will be further personalized with notifications and visualizations.
    ○ Workflow integration (intuitive sequencing of user tasks) will be customized.

## Conclusion

CCNA's COMPASS-ND study leverages the infrastructure of LORIS, an established data management platform with the ability to harmonize, consolidate, and disseminate heterogeneous data types in a user-friendly, and robust fashion. LORIS has been fully customized to the pan-Canadian nature of CCNA, and offers the flexibility to allow for ongoing development as the study matures. This infrastructure also meets the evolving needs of the Canadian data sharing landscape, where CCNA is an exemplar of the successful efforts to consolidate data across the country to accelerate discovery in NDD research.

## Ethics Statement

This study was reviewed and approved by the Research Review Office of the Centre Intégré Universitaire de Santé et de Services Sociaux de Centre-Ouest-de-lÎle-de-Montréal. Participants signed an informed consent form including information on digital storage of collected data. Study is registered at ClinicalTrials.gov with the ID number NCT0340291.

## Author Contributions

ZM and SD contributed to the writing of this paper, contributed to the conceptualization and design of the initiative. VW, HC, and AE contributed to conception and design of the study, contributed to policy. DL contributed to the design of the figures and tables. RA-H, MS-H, and DB contributed to design and technology. JC, CH-B, J-FT, LE, TC, and P-EM contributed to the reading, revision, and approval of the submitted version of the manuscript.

### Conflict of Interest Statement

The authors declare that the research was conducted in the absence of any commercial or financial relationships that could be construed as a potential conflict of interest. The reviewer AI and handling editor declared their shared affiliation at time of review.

## Footnotes

^2^http://www.cihr-irsc.gc.ca/e/46475.html

^3^http://ccna-ccnv.ca

^4^http://loris.ca

^5^http://ondri.ca

^6^http://www.cima-q.ca/en/home

^7^“COMPASS-ND Study.” CCNA. Available online at: http://ccna-ccnv.ca/en/compass-nd-study/ (Retrieved December 11, 2016).

^8^http://truepositivemd.com

^9^http://adni.loni.usc.edu

^10^http://braininstitute.ca/research-data-sharing/brain-code

^11^https://xtxgate.braincode.ca/notify

^12^https://www.discourse.org

^13^Using the Affymetrix UK Biobank Axiom array chip for genetic association analysis at Mt. Sinai Clinical Genomics Centre.

^14^Electroencephalography (EEG).

^15^http://bids.neuroimaging.io/

^16^https://www.ucalgary.ca/esmithresearch/projects/favr

^17^Using data collected in LORIS from the following tools: a) Actigraphy for Quantification of Sleep Architecture and Circadian Irregularity, b) WatchPAT Finger Plethysmography for Quantification of Sleep Apnea, and c) Ambulatory Polysomnography sleep recording.

^18^Scans acquired are for one CCNA and two ONDRI human phantoms. Both studies will be augmented to 3 human phantoms to measure inter-scanner variability. Data will eventually be shared openly.

^19^Brain Imaging Data Structure.

^20^Brainverse is an example.
