# Supplementary material for: National Neuroinformatics Framework for Canadian Consortium on Neurodegeneration in Aging (CCNA)
Source: Front Neuroinform. 2018 Dec 21;12:85. doi: 10.3389/fninf.2018.00085 (PMC6308193; doi:10.3389/fninf.2018.00085)
Supplement: Supplementary file 1 [file Presentation_1.PPTX]

## Slide 1
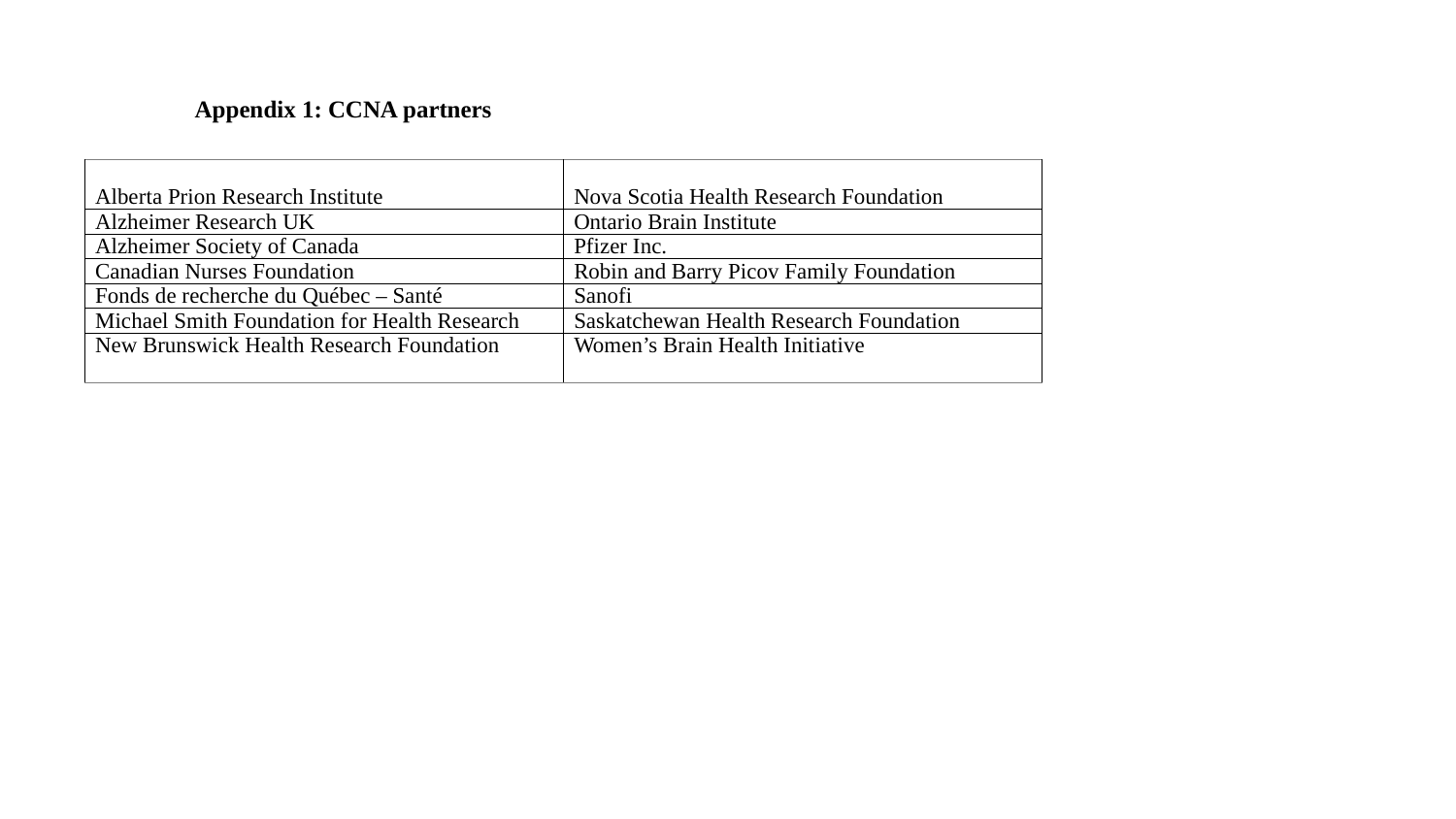

Appendix 1: CCNA partners
| Alberta Prion Research Institute | Nova Scotia Health Research Foundation |
| --- | --- |
| Alzheimer Research UK | Ontario Brain Institute |
| Alzheimer Society of Canada | Pfizer Inc. |
| Canadian Nurses Foundation | Robin and Barry Picov Family Foundation |
| Fonds de recherche du Québec – Santé | Sanofi |
| Michael Smith Foundation for Health Research | Saskatchewan Health Research Foundation |
| New Brunswick Health Research Foundation | Women’s Brain Health Initiative |
