# Supplementary material for: National Neuroinformatics Framework for Canadian Consortium on Neurodegeneration in Aging (CCNA)
Source: Front Neuroinform. 2018 Dec 21;12:85. doi: 10.3389/fninf.2018.00085 (PMC6308193; doi:10.3389/fninf.2018.00085)
Supplement: Supplementary file 2 [file Presentation_2.PPTX]

## Slide 1
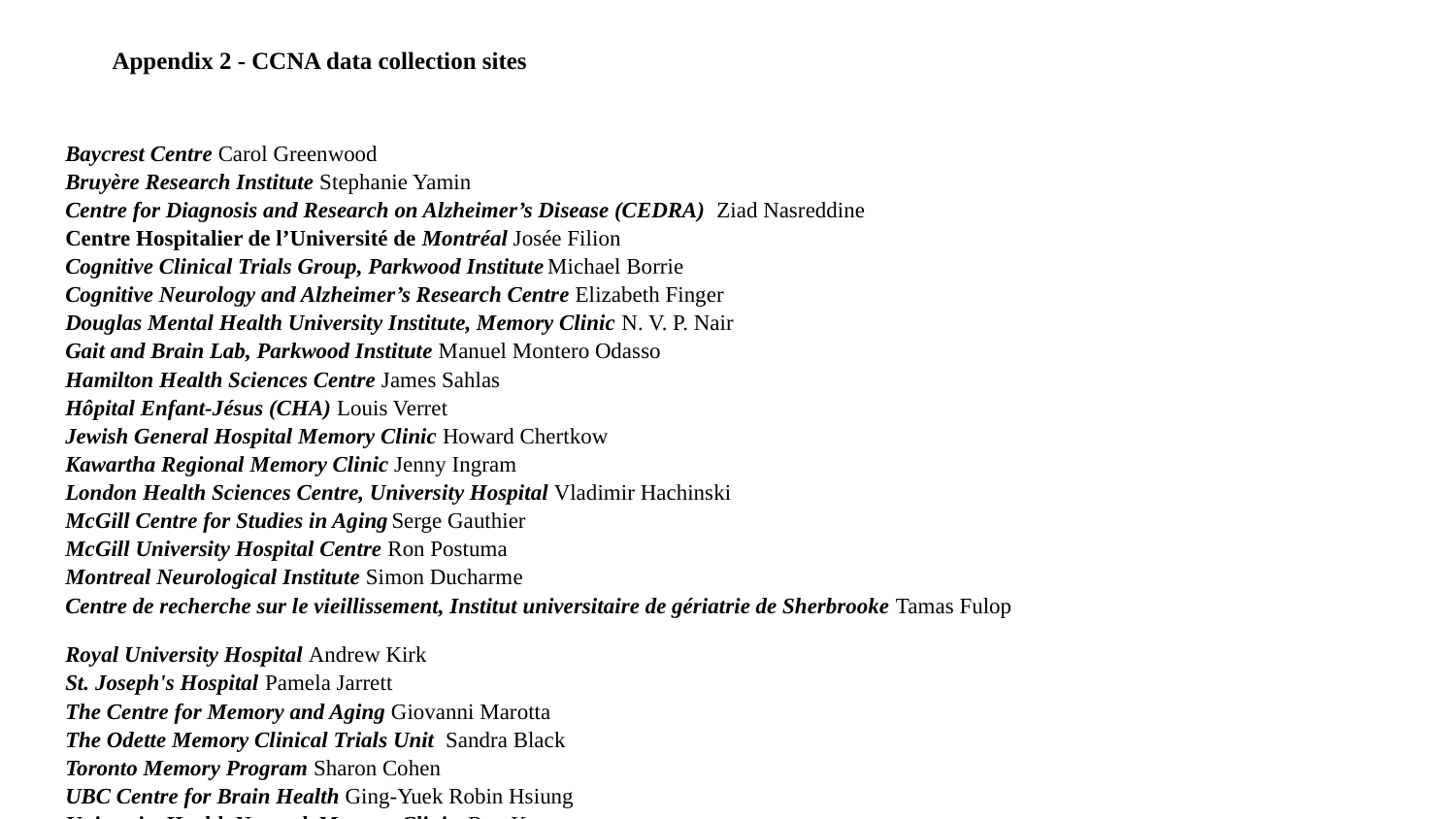

Appendix 2 - CCNA data collection sites
| |
| --- |
| Baycrest Centre Carol Greenwood |
| Bruyère Research Institute Stephanie Yamin |
| Centre for Diagnosis and Research on Alzheimer’s Disease (CEDRA) Ziad Nasreddine |
| Centre Hospitalier de l’Université de Montréal Josée Filion |
| Cognitive Clinical Trials Group, Parkwood Institute Michael Borrie |
| Cognitive Neurology and Alzheimer’s Research Centre Elizabeth Finger |
| Douglas Mental Health University Institute, Memory Clinic N. V. P. Nair |
| Gait and Brain Lab, Parkwood Institute Manuel Montero Odasso |
| Hamilton Health Sciences Centre James Sahlas |
| Hôpital Enfant-Jésus (CHA) Louis Verret |
| Jewish General Hospital Memory Clinic Howard Chertkow |
| Kawartha Regional Memory Clinic Jenny Ingram |
| London Health Sciences Centre, University Hospital Vladimir Hachinski |
| McGill Centre for Studies in Aging Serge Gauthier |
| McGill University Hospital Centre Ron Postuma |
| Montreal Neurological Institute Simon Ducharme |
| Centre de recherche sur le vieillissement, Institut universitaire de gériatrie de Sherbrooke Tamas Fulop |
| Royal University Hospital Andrew Kirk |
| St. Joseph's Hospital Pamela Jarrett |
| The Centre for Memory and Aging Giovanni Marotta |
| The Odette Memory Clinical Trials Unit Sandra Black |
| Toronto Memory Program Sharon Cohen |
| UBC Centre for Brain Health Ging-Yuek Robin Hsiung |
| University Health Network Memory Clinic Ron Keren |
| University of Alberta Richard Camicioli |
| University of Calgary Eric Smith |
| University of Northern British Columbia Jacqueline Pettersen |
| University of Waterloo Laura Middleton |
| Vancouver General Hospital Teresa Liu-Ambrose |
| Vancouver Island Health Authority Alexandre Henri-Bhargava |
| Wilfrid Laurier University Quincy Almeida |
| |
